# Shrimp allergy leading to severe transfusion reaction: A case report

**DOI:** 10.1002/jha2.1021

**Published:** 2024-10-07

**Authors:** Myrthe E. Sonneveld, Sophie J. Bernelot Moens, Jolanda van den Akker, Josephine M. I. Vos, Arjan J. Kwakernaak

**Affiliations:** ^1^ Department of Haematology Amsterdam University Medical Center, University of Amsterdam Amsterdam the Netherlands; ^2^ Amsterdam University Medical Center, University of Amsterdam Amsterdam the Netherlands; ^3^ Sanquin Blood Supply Amsterdam the Netherlands; ^4^ Department of Internal Medicine, Division of Clinical Immunology and Allergy, Department of Nephrology and Transplantation, Amsterdam Institute for Immunology and Infectious Diseases Amsterdam University Medical Center, University of Amsterdam Amsterdam the Netherlands

**Keywords:** allergy, platelet transfusion, transfusion reaction

## Abstract

**Background:**

Transfusion reactions occur at an estimated incidence of 2 per 1.000 transfused products. Anaphylactic transfusion reactions are rarer, and seen in 1 per 10.000 transfusions, and are mostly related to platelet transfusions. Here, we describe a rare cause of a transfusion reaction.

**Case presentation:**

A 19‐year‐old man underwent an allogeneic haematopoietic stem cell transplantation for sickle cell disease and developed an anaphylactic shock following a platelet transfusion, after excluding all common causes. The patient reported a shrimp allergy, and one of the blood/platelet donors had consumed shrimp the day before donation. Elevated levels of specific immunoglobulin E (IgE) directed against shrimp and tropomyosin allergens were found in the patient. Subsequent transfusions were performed with apheresis platelets from selected donors who were instructed to avoid shrimp consumption, and these transfusions were uneventfully.

**Conclusion:**

When a severe transfusion reaction occurs in a patient with a known food allergy, an IgE‐mediated (food‐related) transfusion reaction should be considered after excluding other causes.

## 1 CASE PRESENTATION

A 19‐year‐old man was hospitalized for an allogeneic haematopoietic stem cell transplantion to cure his complicated HbSC sickle cell disease. He received a haploidentical bone marrow transplant from his father, with major blood type incompatibility (A+/B+). Per protocol, he was supported with frequent red blood cell and platelet transfusions (threshold of < 7 g/dL and < 10 × 10^9^/L, respectively, ABO and Rhesus identical, plasma‐reduced) without problems. He had no history of prior blood transfusions reactions. On day 13 post‐transplant, he developed nausea, abdominal pain, and facial edema within minutes after starting a platelet transfusion. This rapidly progressed to cardiopulmonary distress, with a decrease in oxygen saturation to 75%, hypotension (99/55 mmHg) and tachycardia (123/min), and subsequent loss of consciousness. No hives were observed. The transfusion was stopped, and the patient was treated with oxygen therapy (15 L/min), intramuscular adrenaline (0.5 mg), and intravenous antihistamines (clemastine 2 mg). His vital signs normalized immediately, and he regained consciousness. Transfusion‐associated acute lung injury was ruled out based on pulmonary auscultation. The patient had a normal body temperature, and no bacterial contamination was found in the transfusion product. His immunoglobulin A (IgA) levels were normal, and no antibodies against IgA were detected. One hour after the transfusion, his platelet count increased from <10 to 20 × 10^9^/L. No human leukocyte antigen (HLA) or human platelet antigen antibodies (HPA) were detected.

The patient reported experiencing a severe reaction one year earlier after eating shrimp with nausea, abdominal pain, vomiting, angio‐edema, and hives. He was treated with adrenaline and antihistamines. Since then, he avoided all seafood. We hypothesized that the transfusion reaction was IgE‐mediated and possibly related to his shrimp allergy, given the immediate response to adrenaline administration. Unfortunately, tryptase levels were not measured during the reaction. To confirm his shrimp allergy, we measured IgE against shrimp and tropomyosin, which were elevated (9.0 kU/L; ref < 0.34 kU/L and 7.5 kU/L; ref < 0.34 kU/L, respectively). Platelet transfusions typically involve products from five donors. We contacted all donors to inguire about their diets in the days leading up to the donation. One blood donor reported consuming a shrimp cocktail the evening before donating blood. Following this severe transfusion reaction, the patient was successfully transfused with apheresis platelet products until haematologic recovery. We selected blood donors who have not consumed seafood for 10 days prior to donation.

## 2 DISCUSSION

Transfusion reactions are reported in 2 per 1.000 transfused products in the United States [1]. Anaphylactic transfusion reactions are rarer, occuring in 1 per 10.000 transfusions, most often related to platelet transfusions [2, 3]. A platelet transfusion product consists of five buffy coats; which are suspensions of red blood cells, platelets, and plasma from whole blood. After centrifuging, the platelets are separated. An allergic transfusion reaction results from the binding of antibodies in the patient to plasma proteins of the donor or products in the donors' plasma and can be IgE‐ or non‐IgE mediated. IgA deficiency is the most common cause of a (non‐IgE mediated) allergic transfusion reaction.

We found only one case report of a shrimp allergy‐induced transfusion reaction [4]. In that report, a 64‐year‐old male developed angio‐edema and hives after receiving a transfusion with multiple blood products. The patient was sensitized to shrimp, and one of the blood donors had consumed shrimp the night before blood donation. However, IgE against tropomyosin was not reported, and the patient had never experienced a systemic reaction after eating shrimp. Tropomyosin is an immunogenic protein in the cytoskeleton of shellfish and mollusks and is associated with severe systemic allergic reactions [5]. As tropomyosin is resistant to denaturation by heat or degradation by gastrointestinal proteases [6], it can be transmitted through blood transfusion when blood is donated shortly after consumption, potentially causing a severe allergic reaction in an IgE‐sensitized recipient. Some cases describe anaphylactic transfusion reactions provoked by food allergens other than shrimp [7, 8]. Jacobs et al. describe a 4‐year‐old boy with a severe peanut allergy who developed anaphylaxis after a platelet transfusion from a donor who had consumed peanuts shortly before blood donation [9]. The peanut allergen Ara h2 is, like tropomyosin in shrimp, heat‐ and protease‐resistant and can migrate to the bloodstream of the donor and subsequently be transfused, acting as a food allergen in an allergic recipient [6].

A limitation in our case is that we did not measure tryptase levels, nor did we test the transfusion product for shrimp allergens to substantiate the IgE‐mediated etiology of the reaction. It has previously been demonstrated that a haematopoietic stem cell transplantation profoundly reduces allergen‐specific IgE responses, but IgE can be detectable in a small proportion of patients post‐transplantation [10]. Future testing will be performed to evaluate if our patient is cured of his shrimp allergy post‐stem cell transplantation.

## 3 CONCLUSION

In the case of a transfusion reaction, after excluding common causes, an anaphylactic reaction to food allergens in an IgE‐sensitized patient should be considered. Allergy consultation and testing (tryptase and specific IgE) may aid in acquiring the diagnosis. In the case of IgE‐mediated transfusion reaction, the patient can benefit from using apheresis products from donors who avoided consuming the allergen.

## AUTHOR CONTRIBUTIONS

All others contributed to patient care, analysis, and interpretation of the test results. Myrthe E. Sonneveld and Arjan J. Kwakernaak wrote the paper, which was critically revised and approved by all authors.

## CONFLICT OF INTEREST STATEMENT

Coauthor J. Vos is receiving consultancy and advisory board honoraria from Sanofi and Janssen, research support from Beigene and Abbvie/Genmab, and participates in the speakers' bureau for BMS, Sanofi, and Amgen. All honoraria received are directed to the institute. All other authors declare no conflict of interest.

## FUNDING INFORMATION

The authors received no specific funding for this work.

## ETHICS STATEMENT

The authors have confirmed ethical approval statement is not needed for this submission.

## PATIENT CONSENT STATEMENT

The signed consent form was added.

## CLINICAL TRIAL REGISTRATION

The authors have confirmed clinical trial registration is not needed for this submission.

## Data Availability

The data that support the findings of this study are available from the corresponding author upon reasonable request.
